# Tissue Distribution of the MERS-Coronavirus Receptor in Bats

**DOI:** 10.1038/s41598-017-01290-6

**Published:** 2017-04-26

**Authors:** W. Widagdo, Lineke Begeman, Debby Schipper, Peter R. van Run, Andrew A. Cunningham, Nils Kley, Chantal B. Reusken, Bart L. Haagmans, Judith M. A. van den Brand

**Affiliations:** 1 Department of Viroscience, Erasmus MC, Rotterdam, The Netherlands; 2 Institute of Zoology, Zoological Society of London, Regents Park, London, United Kingdom; 3 Institute for Novel and Emerging Infectious Diseases, Friedrich Loeffler Institute, Greifswald, Mecklenburg-Vorpommern, Germany

## Abstract

Middle East respiratory syndrome coronavirus (MERS-CoV) has been shown to infect both humans and dromedary camels using dipeptidyl peptidase-4 (DPP4) as its receptor. The distribution of DPP4 in the respiratory tract tissues of humans and camels reflects MERS-CoV tropism. Apart from dromedary camels, insectivorous bats are suggested as another natural reservoir for MERS-like-CoVs. In order to gain insight on the tropism of these viruses in bats, we studied the DPP4 distribution in the respiratory and extra-respiratory tissues of two frugivorous bat species (*Epomophorus gambianus* and *Rousettus aegyptiacus*) and two insectivorous bat species (*Pipistrellus pipistrellus* and *Eptesicus serotinus*). In the frugivorous bats, DPP4 was present in epithelial cells of both the respiratory and the intestinal tract, similar to what has been reported for camels and humans. In the insectivorous bats, however, DPP4 expression in epithelial cells of the respiratory tract was almost absent. The preferential expression of DPP4 in the intestinal tract of insectivorous bats, suggests that transmission of MERS-like-CoVs mainly occurs via the fecal-oral route. Our results highlight differences in the distribution of DPP4 expression among MERS-CoV susceptible species, which might influence variability in virus tropism, pathogenesis and transmission route.

## Introduction

Middle East respiratory syndrome coronavirus (MERS-CoV) emerged in the human population in 2012 and has been causing multiple outbreaks of human disease, mainly in the Arabian Peninsula^1^. The dromedary camel (*Camelus dromedarius*) has been shown to be the reservoir host for primary human infections^2–8^, although other susceptible animals^9–11^, including bats^12,13^, are suspected also to be hosts for this virus. MERS-like-CoVs have been sequenced from bat samples, mainly from insectivorous bats, but they have not yet been successfully isolated^14–21^. Screening of over 5000 insectivorous bats from Ghana, Ukraine, Romania, Germany, and the Netherlands showed that MERS-CoV-like viruses were detected in 24.9% of *Nycteris* bats and 14.7% of *Pipistrelle* bats^17^.

MERS-CoV uses dipeptidyl peptidase-4 (DPP4) as its receptor to infect its target cells, including bat cells^22^. Analysis of DPP4 sequences from different bat species and *in-vitro* infection studies with various bat cell lines suggested that multiple bat species are susceptible to MERS-CoV^12,21,23^. MERS-like-CoVs probably also use DPP4 as indicated by studies on the Tylonycteris bat CoV HKU4, one of the MERS-like-CoVs^21^. HKU4 uses DPP4 to infect both bat and human cells *in vitro* ^24,25^. It is known that DPP4 is differently distributed in the respiratory tract of humans and other susceptible livestock animals, including dromedary camels^9,26^. DPP4 expression in the nasal epithelium of the camel, llama, and pig allows them to develop upper respiratory tract infection upon intranasal inoculation with MERS-CoV^2,9,26^, while in humans, DPP4 is exclusively expressed in the lower respiratory tract epithelium, which is in line with acute pneumonia being the main clinical outcome of MERS-CoV infection^26,27^. Additionally, the absence of DPP4 expression in the upper respiratory tract epithelium of sheep renders this tissue to be non-susceptible *in-vivo* ^9^. These data indicate that the localization of DPP4 expression in tissues reflects MERS-CoV susceptibility and tropism *in vivo*. The localization of DPP4 expression in bat tissues, however, has not been studied, unlike that in other MERS-CoV susceptible species^9,26^.

Our study aimed to understand the tropism of MERS-like-CoVs in bats by mapping the distribution of DPP4 expression in tissues from four bat species. DPP4 immunohistochemistry staining was performed on tissues collected from two widespread insectivorous bat species in Europe and Asia, the common pipistrelle bat (*Pipistrellus pipistrellus*) and the serotine bat (*Eptesicus serotinus*)^28,29^; and two common frugivorous bat species in Africa, i.e. the Gambian epauletted fruit bat (*Epomophorus gambianus*) and the Egyptian fruit bat (*Rousettus aegyptiacus*)^30,31^. These four bat species were chosen based on their interactions with humans as they roost and forage in the human habitat or serve as a human food source^28–31^. We show that DPP4 localization varies not only among MERS-CoV susceptible species^9,26^ but also between bat species, which may imply variability in MERS-like-CoVs tropism, pathogenesis, and transmission route.

## Results

Immunohistochemistry to detect DPP4 was performed on nose, lung, intestine, kidney, salivary gland, and liver tissues of different bat species: common pipistrelle bat, serotine bat, Gambian epauletted fruit bat (further referred as Gambian fruit bat), and Egyptian fruit bat. The assay was replicated two-three times for each tissue. We have used the same technique to map DPP4 localization in the respiratory tract tissues of human, dromedary camel, sheep, horse, pig, and llama^9,26^. The antibody used in this study recognizes bat DPP4 as was demonstrated in transfection experiments using cloned Pipistrelle bat DPP4^22^. Hematoxylin and eosin staining on subsequent slides of the same tissues from the bats did not show significant histological changes.

**Figure 1:**
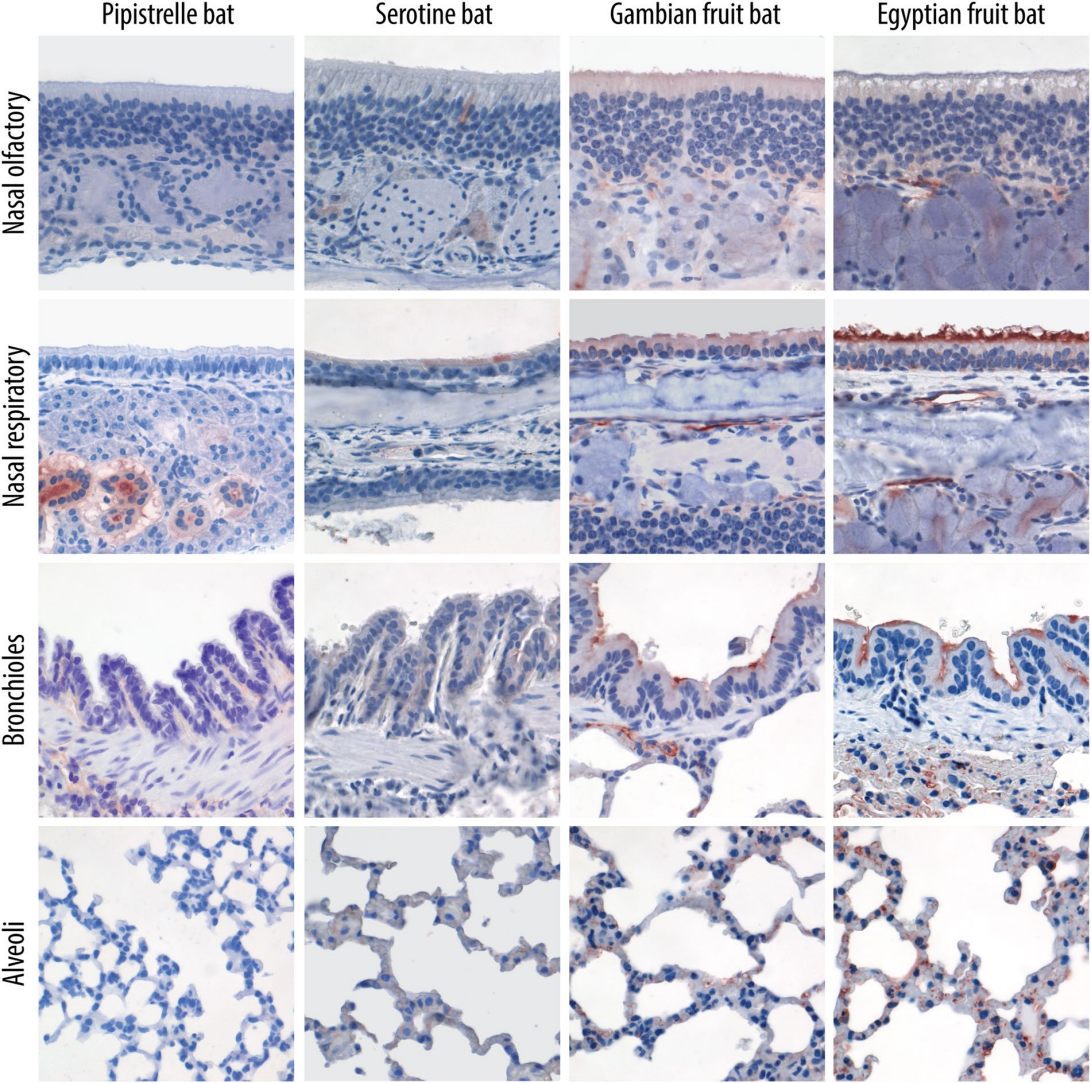
DPP4 expression in the respiratory tract tissues of common pipistrelle bat, serotine bat, Gambian epauletted fruit bat, and Egyptian fruit bat. DPP4 (indicated in red) is expressed in the nasal, bronchiolar and alveolar epithelium of the fruit bats, with limited expression in the epithelium lining the nasal cavity of serotine bats, and not detected in the epithelium lining the respiratory tract of the common pipistrelle bats. Original magnification x400 for all images.

DPP4 was not found in the nasal olfactory epithelial cells of common pipistrelle bat, serotine bat, Gambian fruit bat, or Egyptian fruit bat (Fig. 1). In the nasal tissues of common pipistrelle bat, DPP4 was not detected in the respiratory epithelial cells lining the nasal cavity, but was detected in the epithelial cells lining the ducts of the submucosal glands in this species. In the serotine bat and Gambian fruit bat, multifocal DPP4 expression was detected in a limited number of nasal respiratory epithelial cells. In contrast, in the nasal tissues of the Egyptian fruit bat, DPP4 was prominently detected at the apical surface of the respiratory epithelial cells lining the nasal cavity as well as in glandular and ductular epithelial cells of the submucosal glands. In the lungs of the common pipistrelle and serotine bat, DPP4 was found in the endothelial cells of the capillaries but not in the bronchial, bronchiolar or alveolar epithelial cells. In the Gambian and Egyptian fruit bat, DPP4 was detected in the bronchial, bronchiolar and alveolar epithelial cells as well as in endothelial cells of small blood vessels (Fig. 1).

**Figure 2:**
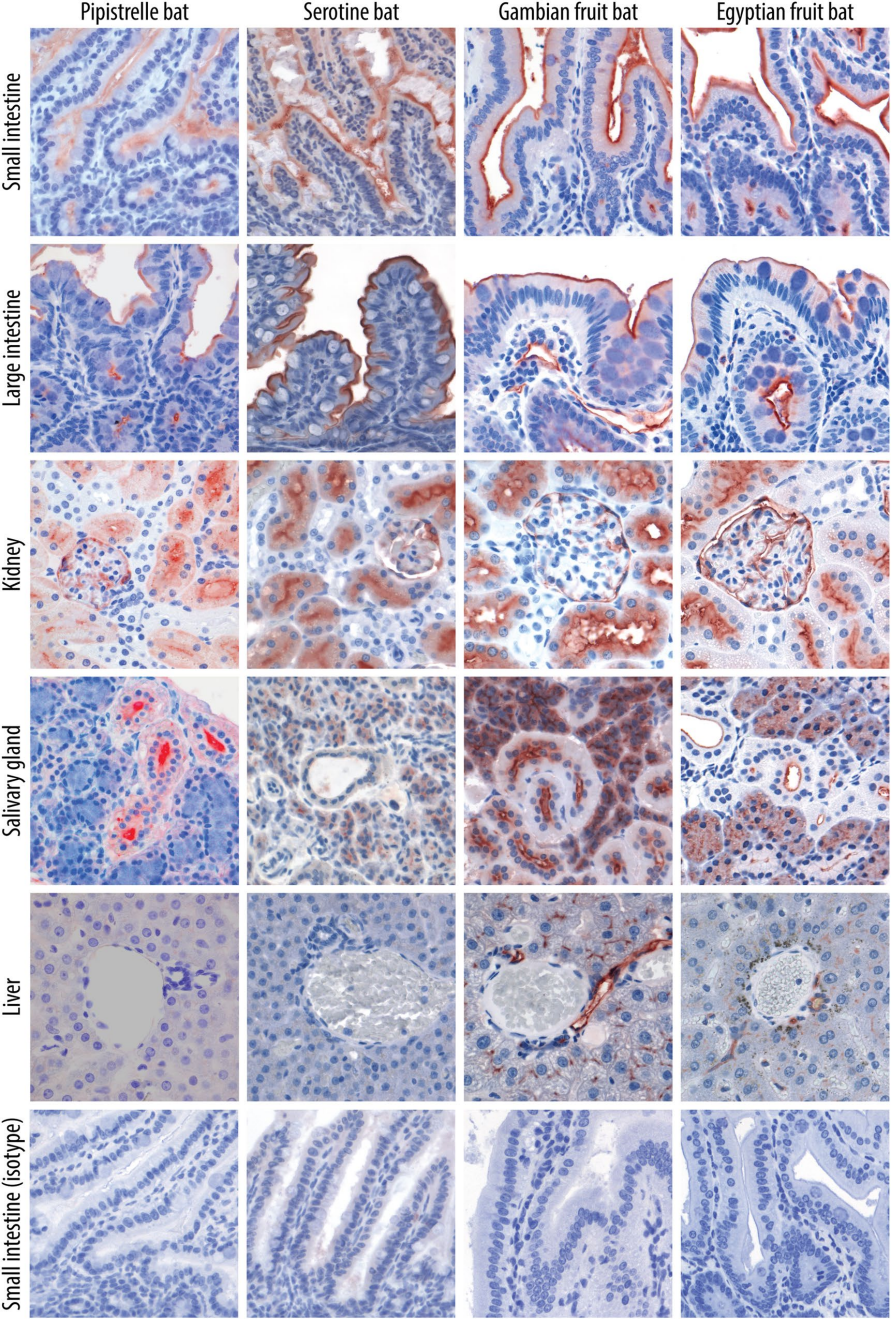
DPP4 expression in the intestine, kidney, salivary gland, and liver tissues of the common pipistrelle bat, serotine bat, Gambian epauletted fruit bat, and Egyptian fruit bat. In all four bat species, DPP4 (indicated in red) is detected on the apical surface of the intestinal epithelium, proximal tubular epithelium of the kidney, and in the salivary glands. Normal goat serum is used as isotype control for each tissue and showed no background signal. Only isotype control staining of the small intestines is shown. Original magnification x400 for all images.

In the intestinal tissues of all four bat species, DPP4 was prominently expressed on the apical surface of both small and large intestinal epithelial cells (Fig. 2). In the kidney of all four bat species, DPP4 was found in glomerular cells, parietal squamous epithelial cells of the Bowman’s capsule, and in the proximal tubular epithelial cells. In the salivary gland of common pipistrelle bat, DPP4 was only detected in the ductular epithelial cells, while in the serotine bat, it was detected in a limited number of glandular epithelial cells. In the Gambian and Egyptian fruit bat, it was detected in both the glandular and ductular epithelial cells of the salivary gland. In the liver of the common pipistrelle bat and serotine bat, DPP4 was present in a limited number of endothelial cells lining the sinusoids. In contrast, in the liver of the Gambian and Egyptian fruit bat, DPP4 was detected in the bile duct epithelial cells, in the endothelial cells of the hepatic arteries, and in the endothelial cells of the sinusoids (Fig. 2). Variation in DPP4 signal and localization were occasionally observed between animals within the same species. In one common pipistrelle bat, the paranasal sinus and pharynx were examined and showed a limited number of DPP4 positive epithelial cells. The results of the DPP4 immunohistochemistry staining were scored qualitatively and summarized in Table 1.

**Table 1:** Overview of DPP4 expression in the tissues of the common pipistrelle bat, serotine bat, Gambian epauletted fruit bat and Egyptian fruit bat.

	Common pipistrelle bat	Serotine bat	Gambian fruit bat	Egyptian fruit bat
**Nose**				
✓Nasal respiratory epithelial cells	−	+/−	+/−	+
✓Nasal olfactory epithelial cells	−	−	−	−
✓Submucosal glands	+	+/−	+/−	+
**Lung**				
✓Bronchiolar epithelial cells	−	−	+/−	+
✓Alveolar epithelial cells	−	−	+/−	+
✓Endothelial cells of the capillaries and small blood vessels	+/−	+/−	+	+
**Intestine**				
✓Small intestinal epithelial cells	+	+	+	+
✓Large intestinal epithelial cells	+	+	+	+
**Kidney**				
✓Glomerular cells	+	+	+	+
✓Parietal squamous epithelial cells of the bowman capsule	+	+	+	+
✓Proximal tubular epithelial cells	+	+	+	+
**Salivary gland**				
✓Glandular epithelial cells	−	+/−	+/−	+
✓Ductular epithelial cells	+	−	+	+
**Liver**				
✓Hepatocytes	−	−	−	−
✓Bile ductular epithelial cells	−	−	+	+
✓Endothelial cells of the hepatic vein	−	−	−	−
✓Endothelial cells of the hepatic artery	−	−	+	+
✓Endothelial cells of the sinusoids	−	+/−	+	+

+, positive detection; +/−, only expressed in a limited number of cells; −, undetected.

**Figure 3:**
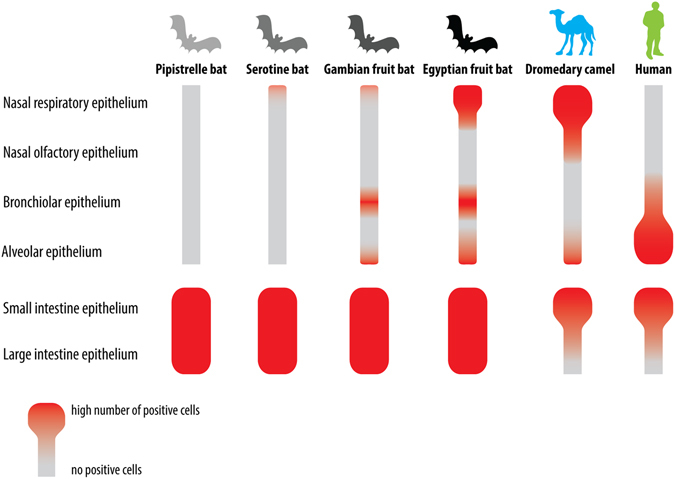
Different distribution of DPP4 expression in the lining respiratory tract and intestinal epithelium of the common pipistrelle bat, serotine bat, Gambian fruit bat, Egyptian fruit bat, dromedary camel, and human. In the common pipistrelle bat and serotine bat, DPP4 is limitedly detected in the respiratory tract and mainly expressed in the intestinal epithelium. In the Gambian and Egyptian fruit bat, DPP4 is found both in the respiratory tract and in the intestinal epithelium. In the dromedary camel, it is expressed in the upper respiratory tract and small intestine epithelium. In the human, it is predominantly expressed in the lower respiratory tract and small intestine epithelium.

In general, our results showed that DPP4 was prominently expressed in the intestine and the respiratory tract tissues of the frugivorous bats, i.e. the Gambian and the Egyptian fruit bat. However, it is limitedly expressed in the respiratory tract tissues of the insectivorous bats, i.e. the common pipistrelle bat and the serotine bat. In the common pipistrelle bat, DPP4 was not detected in the nasal respiratory, nasal olfactory, bronchiolar, or alveolar epithelium, but was abundant on the apical surface of the epithelium lining the small and large intestine. We compared these findings to our previous results on dromedary camel and human tissues^26^. In dromedary camels, DPP4 is strongly detected in the nasal respiratory, tracheal, and bronchial epithelium, while there is limited expression in the alveolar epithelium (Fig. 3). In humans, it is not found in the nasal epithelium and is present mainly in the alveolar epithelium. Additionally, we performed DPP4 staining on intestinal tissues of dromedary camels obtained from a previous study^8^. We found that DPP4 was expressed mainly on the apical surface of the small intestinal epithelium (data not shown), similar to what has been reported for humans^32–35^ (Fig. 3).

## Discussion

The tissue distribution of the MERS-CoV receptor, DPP4, has previously been studied in humans, dromedary camels, and other livestock animals^9,26^. Here, we show that DPP4 is differentially expressed among bat species, especially between insectivorous and frugivorous bats. It is strongly detected in the intestine of the common pipistrelle bat, the serotine bat, the Gambian fruit bat and the Egyptian fruit bat. It is also prominent in the respiratory tract epithelium of the Gambian and Egyptian fruit bat, but expression is limited in that of the common pipistrelle and serotine bat. Given the essential role of DPP4 in the entry of MERS-CoV into cells, these results suggest that MERS-like-CoVs are not likely able to replicate in the respiratory tract in these two insectivorous bats. This is in line with our previous report on MERS-CoV infection experiment in sheep, showing that the lack of DPP4 in the respiratory tract of the sheep was associated with restricted MERS-CoV replication in these animals upon intranasal inoculation^9^. Rather, in these two insectivorous bats, MERS-like-CoVs may preferentially replicate in the gastrointestinal tract. This is partly supported by the fact that viral genomes of MERS-like-CoVs were mainly obtained from faecal and intestinal tissue samples of insectivorous bats^14–20,36^. This intestinal tropism indicates that these viruses transmit mainly through the fecal-oral route. Therefore, future screening of MERS-like-CoVs from insectivorous bats, particularly the common pipistrelle bat, might focus on fecal material, rectal swabs, or intestinal tissues, rather than throat or nasal swabs.

Prominent DPP4 expression in both respiratory tract and intestinal epithelium of the Gambian fruit bat and the Egyptian fruit bat suggests that MERS-CoV is able to replicate in both the respiratory tract and intestine of the fruit bats. These results are in line with the fact that MERS-CoV was able to replicate in the lungs of Jamaican fruit bat (*Artibeus jamaicensis*) upon intranasal and intraperitoneal inoculation^37^. Interestingly, viral RNA could be detected in the rectal swabs of these animals up to day 9 p.i. and infectious virus was also isolated in the duodenum of one of the bats at day 28 p.i.^37^. These data suggest that MERS-CoV infects and replicates in the intestine of these bats, not only in the respiratory tract. MERS-CoV infection in these bats is likely mediated by DPP4, since hamster BHK cells, a non-susceptible cell line, could be infected by MERS-CoV when modified to express Jamaican fruit bat’s DPP4^37^. DPP4 expression in the intestine and respiratory tract of these Jamaican fruit bats, however, was not investigated. Since the Jamaican fruit bat is a new world fruit bat, unlike the Gambian fruit bat and the Egyptian fruit bat, which are old world fruit bats, their genetic difference might influence the variation in DPP4 expression among these species. In contrast to the fruit bats, where DPP4 is expressed throughout the respiratory tract, DPP4 is rarely detected in the respiratory tract tissues of insectivorous bats. This limited DPP4 expression in insectivorous bats might significantly restrict the replication of MERS-like-CoVs in these tissues and minimize the possibility of transmission of these viruses from the respiratory tract.

The limited DPP4 expression in the respiratory tract of the two insectivorous bat species, particularly the common pipistrelle bat, is different from what has been reported for dromedary camels and humans. In humans, DPP4 is merely expressed in the lower respiratory tract, while in the dromedary camels, it is detected in the upper respiratory tract epithelium^26^. This renders humans to develop pneumonia upon MERS-CoV infection, while camels develop upper respiratory tract infection^2,38,39^. In the intestine of both dromedary camels and humans, DPP4 is mainly present in the apical surface of the small intestine epithelium^32–35^. MERS-CoV has been isolated from faecal samples of a naturally infected dromedary camel, which suggests that this virus is able to replicate in the intestinal tract of this species^40^. However, in dromedary camels, the chance of detecting MERS-CoV RNA in faecal samples is much lower than from nasal swabs^40^. We also observed that low amounts of viral RNA are detectable in rectal swabs taken from MERS-CoV- inoculated dromedary camels^2^. While MERS-CoV has not yet been isolated from human faecal samples, low amounts of viral RNA could be detected in stool samples of MERS patients^41^, and several MERS patients have also been reported to suffer from diarrhoea^42–44^. These observations suggest that MERS-CoV replicates in the intestine of both dromedary camels and humans although only to a limited extent. It is currently unclear what factors restrain MERS-CoV replication in the intestinal tract of dromedary camels and humans. The human intestinal tract is protected by a mucus layer, commensal microorganisms, multiple innate and adaptive immune cells^45^. Also, adenosine deaminase (ADA), a natural antagonist of DPP4 that can inhibit MERS-CoV infection *in vitro* ^10^, has also been found in the human intestine. The amount of ADA in the human intestine is four times higher compared to that in the lung^46^. The presence of DPP4 in the intestinal tract of bats suggests an intestinal tropism of MERS-like-CoVs. We also detected DPP4 in the salivary glands and kidneys in all of the bats. *In vitro*, MERS-CoV has also been shown to replicate in primary kidney cell culture derived from common pipistrelle bat^13^. However, there has been no further evidence supporting the susceptibility of these two tissues *in vivo*, nor have there been any reports of MERS-like-CoVs isolated from these two tissues or from bat urine samples. Whether these viruses are transmitted through bat saliva or urine, therefore, is currently unclear.

In general, our study describes the variation in DPP4 distribution among four bat species, with notable differences between insectivorous and frugivorous bats. More importantly, the tissue distribution of DPP4 in insectivorous bats, believed to be one of the natural hosts for MERS-like-CoVs, is different to that in dromedary camels and humans. Our results indicate intestinal tropism of MERS-like-CoVs in the insectivorous bats we examined. The existence of a co-receptor that might influence MERS-like-CoVs tropism and replication in these bats, however, could not be disregarded. CEACAM5 is recently reported as an attachment factor that facilitates entry of MERS-CoV *in-vitro* ^47^. Whether CEACAM5 plays an important role *in-vivo*, particularly in bats, remains to be investigated. *In-vivo* infection experiments are necessary to confirm our findings, but such studies are ethically and technically challenging. Nevertheless, our data are relevant for future monitoring and surveillance of MERS-like-CoVs in insectivorous bats, particularly in the common pipistrelle bat^14,17^, as well as for future efforts to better understand the pathogenesis and transmission of MERS-like-CoVs in their natural host.

## Materials and Methods

Common pipistrelle and serotine bats were found stranded and severely wounded on different occasions, and admitted to an official local bat shelter in the Netherlands. The animals were euthanized by veterinarians due to ethical reasons using officially approved methods. The Gambian fruit bats and three of four Egyptian fruit bats used in this study originated from free-ranging populations in Ghana. The bats were sampled for an unrelated study and this study was approved by the Ethics Committee of the Zoological Society of London (ref. WLE715) and the council for scientific and industrial research in Accra, Ghana. One of the Egyptian fruit bats was obtained from the captive colony at the Friederich Loeffler Institute, Riems, Germany. It had been euthanized due to reasons not related to this study. All methods were performed in accordance with the relevant guidelines and regulations.

After euthanasia, the bats were necropsied and tissues were collected. Parts of the lung, intestine, salivary gland, liver, and kidney were obtained from nine common pipistrelle bats, seven serotine bats, three Gambian fruit bats, and four Egyptian fruit bats. Parts of the noses were obtained from five common pipistrelle bats, six serotine bats, three Gambian fruit bats, and three Egyptian fruit bats. These tissues were all fixed in 10% formalin and embedded in paraffin. The noses were decalcified in 10% EDTA for 9 days before being embedded in paraffin. The formalin fixed paraffin embedded tissues were sectioned (4 μm), deparaffinized, and subsequently hydrated. Citric acid buffer 10 mM pH 6 was used to retrieve antigens. Blocking with normal rabbit serum 5% was performed prior to staining with polyclonal goat IgG anti-DPP4 (R&D systems, Abingdon, UK) in 5 µg/ml concentration. Normal goat serum (MP Biomedicals, Santa Ana, CA, USA) in equal concentration was used as negative control. DPP4 staining was performed at 4 °C overnight. Secondary antibody rabbit anti-goat IgG labeled with peroxidase were applied subsequently in 1:200 dilution for 1 hour at room temperature (Dako, Glostrup, Denmark). The red signal was revealed with 3-amino-9-ethyl-carbazole (Sigma-Aldrich, St. Louis, Missouri, USA) before counterstaining with hematoxylin.

Dromedary camel intestinal tissues were obtained from three different animals sacrificed at day 14 post infection with MERS-CoV in a previous MERS-CoV vaccination experiment^2^. Two of these animals were vaccinated beforehand, while one was not. MERS-CoV was not detected in the intestinal tissues of these animals using PCR, virus titration or immunohistochemistry detecting nucleoprotein of MERS-CoV. Information on DPP4 expression in human respiratory and intestinal tissues was derived from the previous studies^26,33–35^.
